# Efficacy of Use of Triamcinolone Ointment for Clean Intermittent Self Catheterization following Internal Urethrotomy

**DOI:** 10.31729/jnma.3704

**Published:** 2018-08-31

**Authors:** Sunil Regmi, Sunil Chandra Adhikari, Saroj Yadav, Rabin Raj Singh, Ravi Bastakoti

**Affiliations:** 1 Department of Surgery, Morang Sahakari Hospital, Biratnagar, Nepal; 2 Department of Surgery, Koshi Zonal Hospital, Biratnagar, Nepal

**Keywords:** *clean intermittent self catheterization*, *internal urethrotomy*, *Triamcinolone*, *urethral stricture*

## Abstract

**Introduction:**

Internal urethrotomy is recommended for the treatment of urethral strictures shorter than 1.5 cm but has been associated with high recurrence rates. The aim of this study was to evaluate the efficacy of use of triamcinolone ointment for clean intermittent self catheterization in the prevention of urethral stricture recurrence after internal urethrotomy.

**Methods:**

Total of 60 male patients undergoing internal urethrotomy were assigned into two groups and clean intermittent self catheterization was performed using either triamcinolone ointment or a water-based gel for lubrication of the catheter in this randomized clinical trial. Clean intermittent self catheterization regimen was continued for 6 months and patients were followed for 12 months. Urethrocystoscopic evaluation was done 6 and 12 months postoperatively.

**Results:**

The recurrence rates were compared between the two groups. There were no significant differences in patient characteristics and etiology of the stricture between the two groups. There was a 6 (22.22%) recurrence rate in the patients of the triamcinolone group against 13 (46.42%) in those of the control group after the first internal urethrotomy (P=0.04). After second internal urethrotomy, the urethra was stabilized in 5 (83.3%) of the patients in the triamcinolone group and 8 (61.5%) those in the control group (P=0.05). We also found a significant correlation between recurrence and stricture length (P=0.02) but the time to recurrence was not statistically significant (P=0.16).

**Conclusions:**

The use of triamcinolone ointment in patients on CISC regimen after internal urethrotomy significantly decreased the stricture recurrence rate.

## INTRODUCTION

Urethral stricture is one of the oldest known urologie diseases and remains a common problem with high morbidity.^1^ The natural history of disease usually begins with a lesion of the urethral mucosa and infection followed by a scar.^2^ Today most urethral strictures are the result of trauma, inflammatory strictures secondary to gonorrhea infection which were most common in the past are less common now. However, in many cases of anterior urethral stricture disease the etiology remains unknown.^3^

Internal urethrotomy has been recommended for urethral strictures shorter than 1.5 cm, but has been associated with high recurrence rates.^4^ A number of complementary procedures such as indwelling foley catheter, clean intermittent self catheterization (CISC) and urethral stents have been proposed to overcome this problem. In 1972, transurethral use of triamcinolone was proposed by Hebert.^5^ Corticosteroid is used to decrease collagen production, and many studies have shown the efficacy of this treatment.^6^ However, there are not enough studies in the literature to support the use of these agents for internal urethrotomy cases.

We compared the efficacy of using a corticosteroid (1% triamcinolone) for lubrication of the catheter with placebo (water-based lubricant gel) in preventing anterior urethral stricture recurrence in the patients on CISC following internal urethrotomy.

## METHODS

**Figure 1.**
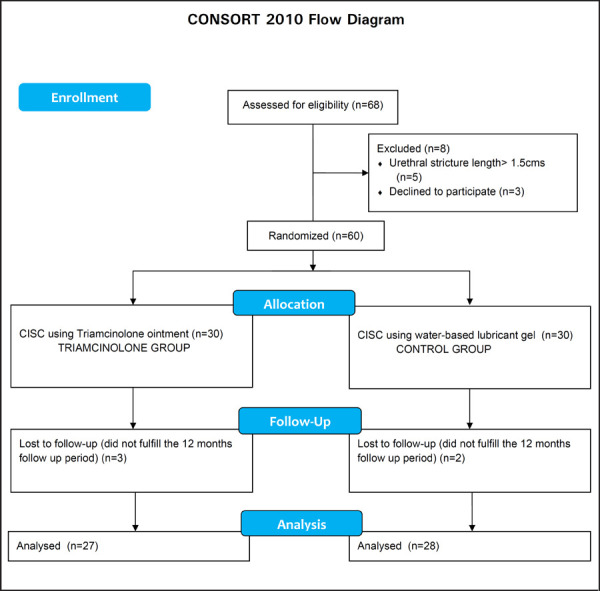
Flow Chart of Patient Selection

Total of 60 male patients with urethral stricture attending the surgical outpatient department of Morang Sahakari Hospital from January 2015 to December 2017 were included in this randomized clinical trial after receiving clearance from IRC of Morang Sahakari Hospital. Preoperative evaluation included a complete history and physical examination, urine culture and retrograde urethrography. We excluded those with urethral strictures longer than 1.5cms. Informed consent was taken from all the eligible patients and they were scheduled for internal urethrotomy and CISC. They were randomly divided into 2 groups using a random table to perform CISC with lubrication by either triamcinolone ointment (triamcinolone group) or a water-based lubricant gel (control group) after internal urethrotomy (Figure 1).

Urethrotomy was performed under direct vision with a cold-knife incision of the stricture using a 21 Fr Storz urethrotome under spinal anesthesia. It was performed by a single surgeon using same technique in all patients. After the procedure, a 16 Fr indwelling catheter was kept for 7 days in all patients. The patients were then instructed to perform CISC by a 16 Fr Nelaton catheter. One peanut size of the triamcinolone ointment was used for lubrication of the catheter. The regimen was tapered over a period of 6 months as shown (Table 1).

**Table 1:** CISC schedule after internal urethrotomy.

Postoperative Time	Catheterization
1^st^ week	Every day
2^nd^ week	Every alternate day
3^rd^ week	Twice in a week
4^th^ week	Once in a week
2^nd^ month	Once in two weeks
3^rd^ to 6^th^ month	Once in a month

All patients were asked to visit at 1, 2, 3, 6, 9 and 12 months after the procedure. They underwent urethrocystoscopic evaluation at 6^th^ and 12^th^ months of follow up. If the patient complained of obstructive symptoms or any difficulty in passing the Nelaton catheter, urethrocystoscopy and internal urethrotomy, if needed was performed.

Statistical analyses were done by Chi square D2 test and Students ‘t’ test using the SPSS software v16 (SPSS Inc, Chicago, USA). P<0.05 was considered significant.

## RESULTS

Three patients in the triamcinolone group and two in the control group were lost to follow-up and were excluded. Analyses were done on the data collected from the records of 27 and 28 patients in the triamcinolone and control groups who fulfilled the 12-month follow-up period after the last internal urethrotomy. There were no significant differences in the baseline characteristics of the patients or the etiology of the stricture between the two groups (Table 2).

**Table 2:** Baseline characteristics of patients in triamcinolone and control groups.

Characteristics	Triamcinolone (n = 27)	Control (n = 28)	P^*^
Mean age (years)	37.2±1.6 (17–70)	36±1.7 (15–75)	0.782
Mean stricture length (cm)	0.93±0.33 ( 0.4–1.5)	1.07±0.32 (0.5–1.5)	0.973
Cause of stricture			
1. Traumatic	12 (44.4%)	14 (50%)	0.768
2. Inflammatory	7 (25.9%)	5 (17.9%)	0.898
3. Others	8 (29.6%)	9 (32.1%)	0.767

* *P< 0.05 was considered statistically significant*

Urethral stricture recurrence was noted in 6 (22.22%) and 13 (46.42%) of the patients in the triamcinolone and control groups, respectively and needed a repeat procedure after the first attempted internal urethrotomy (P = 0.04). The urethra was stabilized in 5 of 6 patients (83.3%) in the triamcinolone group and 8 of 13 (61.5%) in the control group without any stricture recurrence during 12 months of follow-up after second internal urethrotomy (P=0.05). There were no reported febrile urinary tract infection episodes or any other local or systemic complications specific to the use of triamcinolone ointment in our patients.

No statistically significant correlation was observed between stricture recurrence and age of the patient.

However, there was a significant correlation between recurrence and stricture length. Mean stricture length was 10.8±2.2 mm and 8.1±1.6 mm in patients with and without stricture recurrence respectively, (P = 0.02). Although time to recurrence in the triamcinolone group was longer than control group (11.9±3 months vs. 7.4±4.5 months) this difference was not statistically significant (P = 0.16).

## DISCUSSION

Internal urethrotomy has been recommended for urethral strictures shorter than 1.5 cm; however, it has been associated with high recurrence rates.^7^ Urethral stricture recurrence after the first, second, and third internal urethrotomy was reported to be about 50%, 60% to 100%, and 100%, respectively.^8^ Ishigooka and associates mentioned that factors with no influence on recurrent stricture formation included age, etiology, site of the stricture, and duration of indwelling catheterization. On the other hand, stricture length appeared to influence the outcome (P<0.001). Recurrence rate was only 4.4% in short strictures, while it was 42.9% in longer strictures.^9^ Also in our study, there was a significant correlation between stricture length and recurrence (P=0.02).

CISC following internal urethrotomy is an acceptable procedure to reduce the failure rate of the treatment.^10^ Transurethral injection of triamcinolone was addressed by Hebert in 1972.^5^ Many studies have shown clinical improvement of hypertrophic scars after treatment with intralesional corticosteroids. Sharpe and Finney believed that intralesional steroid might be used in many types of strictures, but it was especially useful in cases with strictures in the distal urethra or the meatus, those occurring after radical prostatectomy, and in some cases with one or more urethroplasty procedures.^11^

## CONCLUSIONS

Adding triamcinolone ointment to CISC regimen significantly decreased the recurrence of stricture after internal urethrotomy. Since its use is easy and with low cost, further study involving larger number of patients and longer follow-up is necessary to better elucidate the efficacy and safety of this treatment protocol in patients undergoing CISC after internal urethrotomy.

## Conflict of Interest

**None.**
